# Maternal congenital heart disease: Implications for the next generation

**DOI:** 10.1371/journal.pmed.1005191

**Published:** 2026-07-28

**Authors:** Elisabeth Leirgul, Gottfried Greve

**Affiliations:** 1 Haukeland University Hospital, Bergen, Norway; 2 University of Bergen, Bergen, Norway

## Abstract

In this Perspective, Elisabeth Leirgul and Gottfried Greve discuss emerging evidence that as well as maternal congenital heart disease (CHD) being associated with pregnancy complications, there is also a possible increased risk of long-term developmental challenges in offspring, particularly among children of mothers with severe CHD.

## Teaser

Advances in medical and surgical treatment of congenital heart disease (CHD) now allows most affected individuals to reach adulthood and parenthood. While maternal CHD is associated with pregnancy complications, emerging evidence suggests a possible increased risk of developmental challenges in offspring, particularly among children of mothers with severe CHD.

Congenital heart defects (CHD) constitute the largest group of congenital malformations, with a prevalence of approximately 1% in live births worldwide [1]. Approximately 20% of non-syndromic live births are considered severe [2]. Since the 1990s, advances in medical and surgical treatment of CHD have improved survival considerably, with more than 90% of individuals with CHD now reaching adulthood [3]. As a result, an increasing number of women with CHD are becoming mothers, bringing greater attention to how maternal health, particularly cardiac function, as well as socio‑economic factors, may influence outcomes in their children.

Several important issues arise in this context, including overall parental health—particularly maternal cardiac status—and reproductive outcomes in individuals with CHD. While fecundity does not appear to be markedly impaired [4] neither in women nor men with CHD, those with more severe diseases are less likely to have children [5,6]. Among those who do become parents, however, the number of children is comparable to that of the general population. Importantly, paternal CHD does not appear to increase the risk of adverse neonatal outcomes such as preterm birth or small for gestational age [7], suggesting that maternal factors play a key role in determining pregnancy outcomes.

Pregnancy affects maternal cardiovascular function, and maternal cardiovascular status can, in turn, influence pregnancy outcomes. Several epidemiological studies have reported associations between CHD and pregnancy complications such as fetal growth restriction, pre-eclampsia, preterm birth, and fetal loss [8–10]. Women with complex CHD also face higher risks of maternal cardiovascular complications, including arrhythmias and heart failure. These complications often lead to premature delivery and an increased need for obstetric interventions, including cesarean section [7]. Current clinical guidelines therefore emphasize careful risk stratification and management throughout pregnancy.

Outside the perinatal period, concerns extend to the offspring, including the elevated risk of congenital malformations, particularly cardiac defects [11]. In a Danish study, recurrence risks of CHDs were significantly higher among the offspring of affected women (4.8%) than of affected men (2.7%) [12], suggesting contributions from both genetic factors and the intrauterine environment. In addition, the potential longer-term effects of maternal CHD on offspring are receiving increasing attention. Children born premature or small for gestational age are at increased risk of developmental and functional challenges throughout childhood, including physical health problems as well as cognitive, behavioral, and educational difficulties. However, it is still unknown whether maternal CHD independently contributes to adverse offspring outcomes beyond its association with prematurity and fetal growth restriction, and no validated risk score is currently available to predict long-term offspring outcomes related to maternal CHD. The European Society of Cardiology guidelines include a set of maternal predictors of adverse outcomes in the offspring, including low oxygen saturation (<90%), left-sided obstruction, heart failure, and the use of anticoagulants [13].

Now, a recent study in *PLOS Medicine* by Hossin and colleagues provides important new insights [14]. The authors analyzed a large population-based Canadian study cohort including more than 256,000 children. Development was assessed using teacher-rated Early Development Instrument (EDI) surveys administered in kindergarten around 5–6 years of age. Developmental vulnerability was defined as a score <10th percentile in any two of the five EDI domains: physical health and wellbeing, social competence, emotional maturity, language and cognitive development, and communication and general knowledge. Offspring of mothers with CHD had an increased risk of adverse long-term developmental outcomes at school entry. Children exposed to maternal CHD were 28% more likely to demonstrate developmental vulnerability, with difficulties spanning physical health, social competence, language and cognitive development, and communication skills. The risk was greatest among children of mothers with severe CHD, nearly doubling compared with unexposed children.

**Fig 1:**
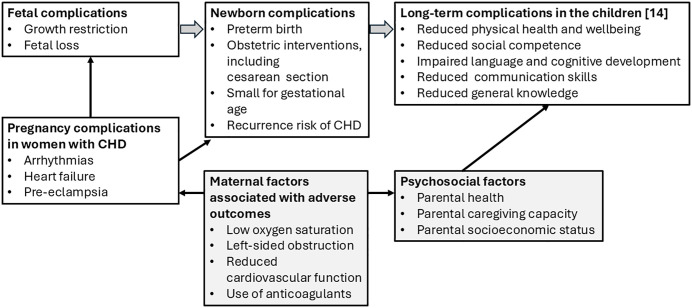
Conceptual framework of how maternal factors may influence pregnancy, neonatal and long-term offspring outcomes, and parental caregiving capacity. Genetic and epigenetic influences are not addressed in this figure.

These findings broaden the focus from survival and immediate perinatal outcomes to wider aspects of child development, indicating that the consequences of maternal CHD may affect multiple domains. They also suggest that maternal CHD severity may be an important determinant not only of pregnancy complications but also of longer-term offspring outcomes (Fig 1).

The mechanisms underlying these associations are likely multifactorial. Preterm birth and fetal growth restriction, which are more common in pregnancies complicated by CHD, may contribute to impaired neurodevelopment (Fig 1). Additional factors may include alterations in the intrauterine environment, genetic susceptibility, and postnatal environmental influences. Psychosocial factors, including parental health and caregiving capacity, may also play a role. Disentangling these pathways remains a major challenge and an important area for future research.

These observations suggest that maternal CHD should be regarded not only as a risk factor for adverse pregnancy outcomes, but also for longer-term offspring health. While preconception counseling and specialized obstetric management remain essential, there is a need to extend care to the postnatal period. Children born to mothers with CHD, particularly those with severe disease, may benefit from closer developmental monitoring and early supportive interventions.

At present, there are no established tools to identify which children are most at risk. Existing risk stratification models focus primarily on maternal cardiovascular outcomes and do not incorporate long-term offspring health. Further research is needed to disentangle the contributions of genetic factors, maternal CHD severity, early-life exposures, and the postnatal social environment. While such work may improve risk prediction, the broader implications extend beyond maternal CHD. Preterm birth and fetal growth restriction are common pathways to developmental vulnerability across many maternal and fetal conditions. Therefore, the greatest public health gains are likely to come from universal developmental screening and follow-up of at-risk children.

In conclusion, the increasing number of women with CHD who reach adulthood and become mothers reflects substantial advances in care. At the same time, it also brings new challenges that extend beyond pregnancy and may affect their children over time. Greater attention to long-term outcomes, rather than short-term obstetric results alone, will be necessary to support both mothers and their children.

## Abbreviations

CHD: congenital heart disease
EDI: Early Development Instrument
